# Protocol for a randomised controlled trial of fetal scalp blood lactate measurement to reduce caesarean sections during labour: the Flamingo trial [ACTRN12611000172909]

**DOI:** 10.1186/s12884-015-0709-7

**Published:** 2015-11-03

**Authors:** Christine E. East, Stefan C. Kane, Mary-Ann Davey, C. Omar Kamlin, Shaun P. Brennecke, On behalf of The Flamingo Study Group

**Affiliations:** Department of Obstetrics and Gynaecology, University of Melbourne & School of Nursing and Midwifery, Monash University, Parkville, 3052 Australia; Department of Perinatal Medicine, Pregnancy Research Centre, The Royal Women’s Hospital, The University of Melbourne, Parkville, 3052 Australia; Department of Obstetrics and Gynaecology, The University of Melbourne, Parkville, 3052 Australia; Department of Perinatal Medicine, The Royal Women’s Hospital, Parkville, 3052 Australia; Judith Lumley Centre (formerly Mother & Child Health Research), La Trobe University, 215 Franklin Street, Melbourne, VIC 3000 Australia; Neonatal Services, Royal Women’s Hospital, Parkville, 3052 Australia

**Keywords:** Caesarean section, Fetal hypoxia, Cardiotocography, Electronic fetal monitoring, Fetal scalp blood sampling, Lactate, Obstetric labour complications

## Abstract

**Background:**

The rate of caesarean sections around the world is rising each year, reaching epidemic proportions. Although many caesarean sections are performed for concerns about fetal welfare on the basis of abnormal cardiotocography, the majority of babies are shown to be well at birth, meaning that the operation, with its inherent short and long term risks, could have been avoided without compromising the baby’s health. Previously, fetal scalp blood sampling for pH estimation was performed in the context of an abnormal cardiotocograph, to improve the identification of babies in need of expedited delivery. This test has largely been replaced by lactate measurement, although its validity is yet to be established through a randomised controlled trial. This study aims to test the hypothesis that the performance of fetal scalp blood lactate measurement for women in labour with an abnormal cardiotocograph will reduce the rate of birth by caesarean section from 38 % to 25 % (a 35 % relative reduction).

**Methods/Design:**

Prospective unblinded randomised controlled trial conducted at a single tertiary perinatal centre. Women labouring with a singleton fetus in cephalic presentation at 37 or more weeks’ gestation with ruptured membranes and with an abnormal cardiotocograph will be eligible. Participants will be randomised to one of two groups: fetal monitoring by cardiotocography alone, or cardiotocography augmented by fetal scalp blood lactate analysis. Decisions regarding the timing and mode of delivery will be made by the treating team, in accordance with hospital protocols.

The primary study endpoint is caesarean section with secondary outcomes collected from maternal, fetal and neonatal clinical course and morbidities. A cost effectiveness analysis will also be performed. A sample size of 600 will provide 90 % power to detect the hypothesised difference in the proportion of women who give birth by caesarean section.

**Discussion:**

This world-first trial is adequately powered to determine the impact of fetal scalp blood lactate measurement on rates of caesarean section. Preventing unnecessary caesarean sections will reduce the health and financial burdens associated with this operation, both in the index and any future pregnancies.

**Trial registration:**

Australian New Zealand Clinical Trials Registry ACTRN12611000172909

## Background

There is currently an epidemic of caesarean sections performed in Australia and overseas [1]. Although many caesarean sections are performed for concerns about fetal welfare, the majority of babies are shown to be well at birth, meaning that the operation, with its inherent short- and long-term risks, could possibly have been avoided, without compromising the baby’s health. This project is a randomised trial of fetal scalp blood sampling for lactate measurement during labour, with a view to reducing the caesarean section rate for apparently non-reassuring fetal status. The trial proposal derives from the Cochrane systematic review of intrapartum fetal scalp blood sampling for lactate measurement [2], which highlighted the need for a randomised trial of its effectiveness.

It is imperative that a randomised controlled trial of lactate measurement be conducted so that potentially unnecessary caesarean sections can be avoided, that may otherwise harm, rather than help, mothers and their babies. As such, this will be a world-first trial.

### The burden of disease associated with caesarean sections

Despite the evidence that caesarean section rates above 15 % seem to do more harm than good [3], countries such as Australia, the United States of America and China report alarmingly high rates of 27-50 % [1, 4, 5]. The Australian caesarean section rate of 32 % translates to over 95,000 women having this surgery per year, with over 39,000 of these occurring following the onset of labour [4].

Risks that are increased with a caesarean birth include anaesthetic risks, maternal infection, venous thromboembolism, haemorrhage [6, 7] and intensive care unit admissions for babies with breathing difficulties [8]. Having had a caesarean section delivery increases risks in subsequent pregnancies of having further caesarean sections, stillbirth, rupture of the uterus, hysterectomy and increased bleeding due to the placenta implanting over the scar [9–11]. These concerns must be balanced against the need on occasions to urgently deliver a sick baby who would be compromised by remaining in the uterus.

A common indication for caesarean sections following the onset of labour is the identification of a non-reassuring fetal heart rate pattern. The chief investigator of this study has previously found that 38 % of Australian labouring women, where a non-reassuring fetal heart rate pattern had been identified, proceeded to delivery by caesarean section if no further testing of fetal well-being was performed (i.e. for pH or lactate). On the other hand, the caesarean section rate was 10 % when such testing was undertaken [12].

### Cardiotocography assessment of fetal well-being during labour

Cardiotocography (CTG), which detects and records the fetal heart rate by Doppler along with uterine contractions, was introduced into clinical practice in the 1960s with the aim of improving perinatal outcomes by improving intrapartum fetal welfare surveillance. A recent clinical audit revealed that 71 % of labouring women have continuous CTG in labour at the Royal Women’s Hospital, Melbourne. Fetal heart rate patterns can be classified in a number of ways. These include: (i) normal/reassuring; and (ii) when non-reassuring, a range of terms including non-reassuring, suspicious, atypical, abnormal, pathological or ominous. These classifications are based on the fetal heart rate, its variability and the presence of accelerations or decelerations, compared with the occurrence of uterine contractions. Several groups have published guidelines in an attempt to provide uniformity of interpretation [13–16]. Consistent with these guidelines and with the review team’s clinical practice, this protocol generally refers to “non-reassuring fetal status or abnormal /CTG/patterns”, rather than the term “fetal distress”, which is sometimes used inappropriately to refer to CTGs that do not meet normal/reassuring status [17]. Reassuring patterns require no specific action. Non-reassuring patterns are identified in approximately 15 % of those with CTG monitoring [18] and may prompt clinical actions ranging from simple manoeuvres, such as maternal position change, improved maternal hydration, through to expedited birth of the baby by caesarean section, forceps or vacuum. These actions aim to prevent or minimise hypoxia in the fetus.

### Compensation mechanisms for decreased oxygen supply: can the intrapartum CTG identify the baby’s response?

Non-reassuring CTG patterns may reflect adaptations made by the individual fetus to decreases in oxygen supply. Inadequate oxygen supply results in anaerobic metabolism of glucose, which leads to metabolic acidosis and the production of lactate. Low pH is a combined measure of both metabolic acidosis (including base deficit) and the more labile component, respiratory acidosis.

The differences in individual fetal responses to a decrease in oxygen (and therefore differences in fetal heart rate changes) mean that the positive predictive value of non-reassuring CTG for adverse outcome is low, although the negative predictive value of a reassuring pattern is high [18]. This means that a normal CTG almost always indicates reassuring fetal status, while a non-reassuring CTG on its own significantly overestimates the occurrence of babies that would have a poor perinatal outcome if birth were delayed. These features, combined with marked inter-observer variation in CTG interpretation by midwives and doctors, result in high caesarean section rates for non-reassuring fetal status in many hospitals [19, 20].

### Fetal scalp blood sampling

Collection of a small capillary blood sample from the fetal scalp (a fetal blood sample) for blood gas analysis has been used in clinical practice since the 1960s to assist in intrapartum fetal welfare evaluation [21]. Following identification of one abnormal or two non-reassuring features on the CTG, the National Institute for Health and Care Excellence in the United Kingdom recommends fetal blood sampling (FBS) for pH or lactate estimation, with a published action algorithm based on the result [14]. Following rupture of the amniotic membranes and at cervical dilatation greater than or equal to approximately 3 cm, an amnioscope is placed vaginally to allow adequate visualisation of the fetal head. A small sample of blood is then taken from the fetal scalp. This procedure may be uncomfortable and intrusive for the mother and is invasive to the baby. Rare complications include fetal scalp infection and haemorrhage [22, 23] and it is contra-indicated when the mother is known to have HIV or viral hepatitis, or where there is suspicion of a bleeding tendency in the fetus [23, 24].

Traditionally, such testing has required approximately 30 to 50 microlitres of blood, which is often difficult to obtain [25]. Even when the clinician is able to collect this quantity of blood, samples are frequently rejected by the testing equipment due to contamination with air or amniotic fluid. Some equipment can analyse for other components, such as lactate. Fetal lactate testing equipment requiring a much smaller blood volume (as little as 5 microlitres) is now available [26].

### Fetal blood sampling for pH estimation: what is the supporting evidence for use?

The Cochrane systematic review of intrapartum CTG for fetal welfare [27] reported that:

- In a meta-analysis of five RCTs enrolling 18,700 mother/baby pairs, the relative risk (RR) for caesarean section was 1.96 (95 % confidence intervals [CI] 1.23 to 3.09) for the continuous CTG group compared with intermittent auscultation of the fetal heart.
- Meta-analysis of six RCTs (*n* = 15,000) resulted in an RR of 1.5 (95 % CI 1.10 to 2.06) for caesarean section when fetal well-being was monitored by CTG and fetal scalp blood pH sampling, versus intermittent auscultation.
- Only one of the published RCTs compared outcomes following allocation to CTG + FBS (pH) or CTG alone: four of 229 women in the CTG + FBS group underwent caesarean section, compared with 41 of 230 in the CTG-only group, yielding a relative risk of 0.64 (95 % confidence intervals 0.40 to 1.00) [28]. There were no differences in any immediate infant outcomes (Apgar scores, cord blood gases, neonatal death, neonatal morbidity, nursery course).

These trials and the systematic review influenced obstetric opinion toward recommending FBS when a non-reassuring CTG is identified during labour, for example, the National Institute for Health and Care Excellence in the United Kingdom [14] clinical practice guidelines, which are closely aligned with Australian clinical practice. Not all countries follow this line of persuasion, however, with FBS for pH estimation being uncommon in the United States of America, for example [29].

The use of pH estimation in Australia has declined, largely replaced by the availability of hand-held bedside lactate testing units, which require much smaller blood samples than for pH.

### Fetal blood sampling for lactate measurement: an alternative to pH estimation

A fetal scalp blood sample for lactate measurement (FBSLM) taken within 60 min prior to birth correlates well with umbilical arterial and venous lactate, pH and base excess measured following birth [30]. Umbilical arterial (UA) lactate values correlate well with UA pH and base deficit values [30, 31].

Allen et al. [32] reported that fetal scalp lactate values greater than or equal to 4.2 mmol/L offered the best sensitivity and specificity for detecting clinically important outcomes. Kruger et al. [25] retrospectively examined the predictive values of 326 simultaneous fetal scalp blood estimations of lactate and pH for Apgar scores, umbilical arterial pH, umbilical arterial base deficit and neonatal encephalopathy. Cut-off values were the 75th centile for lactate (4.8 mmol/L) and the 25th centile for pH (7.20). The area under the receiver operating characteristic (ROC) curve was significantly greater for lactate than for pH in predicting neonatal encephalopathy and low Apgar score [25]. These findings and evidence from animal studies of the effects of lactate on brain tissue [33, 34] suggest that lactate estimation may be a better predictor of severe neonatal morbidity than pH.

### Lactate values that prompt clinical intervention

Because different lactate values can be simultaneously recorded from different lactate measurement systems [35–37], the cut-off lactate value selected to prompt clinical action must be considered specifically for the lactate meter in use [2, 37]. The available data support a lactate value greater than 4.8 mmol/L as a cut-off value for intervention when measured with the Lactate Pro (Arkray, Kyoto, Japan) [38], which is used at the RWH and included the RWH Clinical Practice Guidelines [39].

### Intrapartum lactate values

Observational studies suggest that fetal lactate concentrations are constant during the first stage of labour in the absence of hypoxia, and that intrapartum lactate measurements are better than estimations of pH for the prediction of severe neonatal morbidity [25, 33, 34, 40, 41]. Nordström [42] found that, on average, maternal lactate rose by 2 mmol/l per 30 min in the second stage of labour. Fetal lactate concentrations correlate positively with the duration of active pushing at a rate about half that of the maternal lactate (1 mmol/L per 30 min) [42]. The question of whether this lactate rise is driven by fetal hypoxia or derived from the mother was investigated by studying the arterio-venous lactate difference at birth. They found that the main contributor to the fetal lactate increase is the fetus itself, especially with prolonged second stage [42]. This is also supported by animal studies [43]. These findings suggest that fetal scalp lactate measurements remain an appropriate indicator of fetal hypoxia in second stage, particularly, for example, when the fetal head is relatively high in the mother’s pelvis and a dense epidural analgesia is in place that limits the mother’s ability to bear down and give birth to the baby in a short timeframe (i.e. minutes). The clinical appropriateness of performing a FBSLM in response to abnormal fetal heart rate monitoring in active/advanced second stage warrants consideration, where the option of assisting the birth with vacuum or forceps would be a reasonable option, achievable within minutes, rather than delaying this intervention to take a blood sample.

### Appraising the evidence for lactate measurement in labour: Systematic review

There is only one published systematic review of the clinical effectiveness and risks of fetal scalp lactate sampling used to assess fetal well-being in labour, in the presence of a non-reassuring CTG [2]. Only two randomised trials [38, 44] compared outcomes following fetal scalp blood sampling for pH or lactate measurement. Fetal scalp blood lactate estimation was more likely to be successfully undertaken than pH, with fewer scalp incisions and results available within 60 s. Larger blood volumes and longer test times were required for pH estimation. No randomised trials were identified that compared the use of CTG-only with CTG and lactate, although one ongoing trial has been identified (The SCALP trial [45]) during the process of updating the published Cochrane systematic review. This creates an untenable dilemma. Lactate measurement has essentially replaced pH estimation clinically, but there is no evidence from randomised trials and systematic reviews supporting its use. The value of using lactate measurements in labour without this evidence is uncertain, given the contribution that pH estimation has previously been shown to make for avoiding caesarean birth [46, 47].

### The need for a randomised trial of lactate for assessment of fetal well-being

It is proposed that undertaking this randomised trial is timely and mandatory because:

- Caesarean section following onset of labour is a major contributor to the alarmingly high overall caesarean section rate.
- Caesarean section increases maternal mortality and morbidity in the index and subsequent pregnancies.
- Caesarean section may increase fetal/neonatal morbidity in the index pregnancy.
- Fetal blood sampling following identification of a non-reassuring CTG was previously done for pH estimation and was considered the best option to reduce the risk of unnecessary caesarean sections in many clinical settings.
- Lactate measurement has replaced pH estimation as the test of choice by FBS in contemporary Australian clinical practice and also in many overseas centres.
- Although lactate measurement is possible and the technology readily available, it is not widely practiced in Australian and many overseas maternity centres – that is, a state of equipoise exists.
- There currently exists no RCT data establishing the benefits and risks of FBSLM in labour.
- There is therefore an urgent need for evidence about the benefits and risks of lactate measurement when a non-reassuring CTG has been identified in labour, to enable childbearing women and their clinicians to make an informed choice for mode and timing of birth.

## Methods/Design

### Aim

The primary aim of this randomised trial is to determine whether the addition of FBSLM during labours complicated by a non-reassuring fetal heart rate cardiotocography trace reduces the risk of birth by caesarean section, compared with monitoring by CTG alone.

### Hypothesis

When a woman in labour has a non-reassuring CTG, she has a 38 % chance of delivery by caesarean section [12]. We hypothesise that the addition of FSBLM will reduce this rate from 38 % to 25 %, which is a 35 % relative reduction.

### Study design

This randomised controlled trial conforms to Consort Statement standards [48] and is informed by the investigator team’s experience in the evaluation of technology for fetal welfare assessment [49–52]. The nature of the RCT necessitates non-blinding of clinicians and participants. The trial has been registered with the Australian and New Zealand Clinical Trials Register (registration number ACTRN12611000172909), and has been approved by the Human Research Ethics Committee of the Royal Women’s Hospital, Victoria, Australia – the single site in which the trial is to be conducted (Project 11/56).

### Inclusion criteria

- Abnormal fetal heart rate trace in labour not improved with conservative measures (maternal position change, correction of maternal hypotension, decrease or cessation of oxytocin infusion) (Table 1)

**Table 1:** Abnormal CTG trace eligible for further evaluation by fetal scalp blood sampling

Consistent with hospital, national and international guidelines [14, 16, 39] an abnormal CTG trace eligible for further evaluation by FBS is defined as:	
One or more of	● Baseline FHR <100 beats per minute (bpm) or >170 bpm
	● Variability absent or <3 bpm
	● Prolonged deceleration (>90s and <5 min)
	● Late decelerations
	● Complicated variable decelerations
Both of	● Baseline FHR between 100-109 or 161-170
	● Baseline FHR variability 3-5 bpm for > 40 mins
- Ability to give informed consent in English
- Singleton pregnancy
- Gestation ≥ 37 weeks
- Cephalic presentation
- Cervical dilatation ≥ 3 cm
- Ruptured amniotic membranes

### Exclusion criteria

- Planned caesarean section
- Known viral infections where FBS is contraindicated (e.g. hepatitis, HIV)
- Situations requiring immediate delivery, e.g. significant intrapartum haemorrhage, cord prolapse, etc.
- Known significant fetal anomaly or bleeding disorder

### Trial entry

To maximise participation, women will be recruited to this trial by way of both a conventional ‘opt-in’ approach, and a novel ‘opt-out’ strategy implemented following release in March 2014 of the revised National Health and Medical Research Council’s statement on ethical conduct in human research [53], which provided a framework for the use of opt-out consent.

#### Opt-in consent

Using verbal and written approaches, women planning vaginal birth will be informed of the trial during their antenatal visits, focusing specifically on third trimester hospital clinic visits. If an abnormal FHR traces emerges during labour and the woman is able to comprehend the study, she will be approached to participate.

#### Opt-out consent

From August 2014, eligible women booked and receiving antenatal care at the Royal Women’s Hospital will be provided with written information about the trial at various gestations, including the fact that both using and not using lactate measurement are both common strategies at RWH and are accepted as appropriate by senior clinical staff. The women will be advised that, if they subsequently meet the inclusion criteria and none of the exclusion criteria during labour, they will be part of the trial unless they let the clinical or research staff know that they wish to opt-out. They can let the staff know in a number of ways: verbally, by telephone or email. This information is then recorded in the woman’s medical record and in a register, maintained by and accessible to the appropriate clinicians and researchers. Both opt-in and opt-out consenting processes will continue from August 2014.

### Randomisation and group allocation

A researcher not otherwise involved with the trial will use computerised sequence generation with permuted block randomisation, stratified for parity (nulliparous, parous), to develop the randomisation sequence that will be applied through sequentially numbered, sealed, opaque envelopes. Those women whose CTGs meet the inclusion criteria will be randomised in a 1:1 ratio to the CTG-only group or the CTG + lactate group. Clinical care during labour and birth will continue with the woman’s obstetric and midwifery team. Neonatal care will be the provided by the neonatal team, according to national resuscitation guidelines.

#### Both groups

The hospital’s clinical practice guideline for CTG management will be followed for measures to alleviate umbilical cord compression and/or improve placental blood flow to the fetus, such as maternal position change, correction of hypotension or discontinuation of an oxytocin infusion. Umbilical venous and arterial cord blood will be collected at birth for blood gas analysis.

#### CTG-only group

Following identification of an eligible CTG (Table 1) and randomisation to the CTG-only arm, monitoring of fetal well-being will continue. No FBS will be taken, even when a non-reassuring CTG persists despite measures for improving the CTG. Timing of and progress to normal birth, operative vaginal birth or caesarean section, including for the indication of non-reassuring fetal status, will be at the discretion of the clinicians in consultation with the labouring woman.

#### CTG + lactate group

Following identification of an eligible CTG (Table 1) and randomisation to the CTG + lactate arm, if a non-reassuring CTG persists despite measures for improving the CTG, the timing and options for delivery will incorporate a further evaluation of fetal well-being. Fetal blood sampling for lactate measurement will be undertaken, with resulting actions as per Table 2 (developed from the available evidence and aligned with the Clinical Practice Guideline of the RWH [39]).

**Table 2:** Clinical management for lactate results in the CTG + lactate group

Lactate (mmol/L)	Action
<4.0 mmol/L	Repeat FBS in 1 hour if the FHR abnormality persists
4.0-4.8 mmol/L	Repeat FBS within 30 minutes or consider expediting the birth if rapid rise since last sample
>4.8 mmol/L	Urgent delivery indicated

### Data collection

Baseline demographic and characteristic data will be abstracted from the participants’ hospital records by the clinical research midwives. In keeping with the requirements of the Consort Statement [48], the Clinical Research Midwives will record the number of women potentially eligible for enrolment in the RCT, the reasons for non-enrolment (e.g. laboured too quickly) and limited data on non-enrolled women that are routinely collected for clinical audit, for example, mode of birth, parity, Apgar scores, to achieve representative enrolment. The primary and clinical secondary outcomes will be abstracted from the participants’ medical records by the Clinical Research Midwives.

#### Women’s experiences

We developed a questionnaire of women’s experiences with labour, monitoring and research specifically for our fetal oximetry trial, drawing on key concepts from experts and peer-reviewed literature [51]. The Cronbach’s alpha (0.83 in each of the study groups) and item-total correlation (*p* < 0.001) supported the reliability and validity of the questionnaire. We will use the nine questions of the ten in the original survey [51] that are relevant to this lactate trial. These questions will seek a rating of “poor”, “fair”, “good” or “excellent” in regard to three domains:

(i) labour
(ii) monitoring of fetal well-being during labour and
(iii) participation in the research.

Provision will also be made for free-text comments. The questionnaire will be administered by the research midwives. It will be returned via the internal hospital mail if completed prior to discharge. If the woman has been discharged without being given a questionnaire, we will contact her by phone or mail to send a questionnaire and postage paid envelope for its return. We will follow women up with one phone call or letter if the questionnaire is not returned.

#### Economic analysis

An economic analysis will be undertaken using cost-effectiveness analysis (CEA) to compare the cost and effectiveness of the experimental clinical strategy: adding lactate estimation to conventional CTG monitoring, versus conventional CTG alone. This method determines the “price” of the additional outcome purchased by changing from one practice (CTG-only) to the alternative strategy (CTG + lactate). The CEA will examine change in resource use for the primary endpoint: caesarean section. Costs for treatment related expenses will include direct medical costs, such as fetal monitoring procedures, equipment, mode of birth and postnatal stay. Given the uniformity of the treatment environment in which the study will be undertaken, we consider a limited perspective of costs from the viewpoint of the health service, in which we already have experience, will be appropriate for this evaluation [52, 54, 55].

### Study endpoints

#### Primary study endpoint: caesarean section

The Cochrane systematic review of lactate measurement in labour identified the caesarean section rate as a key endpoint for the evaluation of this form of fetal welfare assessment [2].

#### Secondary study endpoints

1. Maternal endpoints, identified in the Cochrane systematic review [2]:
  - Total operative birth (forceps + vacuum + caesarean section)
  - Total operative vaginal birth (forceps + vacuum)
  - Normal vaginal birth
  - Caesarean section specifically for the indication of non-reassuring fetal status
  - Assisted vaginal birth (forceps or vacuum) for non-reassuring fetal status
  - Maternal satisfaction with fetal monitoring in labour
  - Maternal length of hospital stay
  - Fetal/neonatal endpoints
    The fetal oximetry trial [50] demonstrated equivalence in fetal and neonatal outcomes when comparing the use of CTG with fetal oximetry added to CTG. This is relevant, because although poor outcomes resulting from hypoxia are rare, they are important. For this reason, our team of investigators includes experienced neonatologists. Based on recommendations for definitions of serious outcomes by the Australian New Zealand Neonatal Network and expert recommendation for important measures of morbidity at and beyond term [56, 57], we will record:
    - Composite fetal/neonatal endpoint: Death or serious outcome for the infant: includes one or more of fetal death after trial entry; death of a liveborn infant prior to hospital discharge; neonatal encephalopathy (stages II/III) [58]; Apgar score <4 at 5 min; care in neonatal intensive care unit >96 h.
    - Other neonatal morbidity outcomes will include: individual components of the composite fetal/neonatal outcome; neonatal encephalopathy (stages I, II, III) [58]; Apgar score <7 at 5 min; umbilical cord arterial and venous and first neonatal blood gas (within two hours of birth) pH, base deficit and lactate; resuscitation by bag and mask ventilation, intubation, external cardiac massage and/or adrenaline; admission to neonatal intensive care; neonatal length of hospital stay.
    - Economic: Cost-effectiveness of fetal monitoring
    - Considerations related to fetal scalp blood lactate measurement only: We will also record the success rate of fetal scalp blood sampling for fetal lactate and fetal scalp laceration or infection requiring treatment.

### Sample size calculations

Data from the systematic review of CTG + FBS trials and observational data from Australian hospitals support a conservative estimate of up to a 40 % relative risk reduction in caesarean sections when FBS is added to CTG monitoring [12, 27, 28]. Based on a conservative 35 % relative reduction in Caesarean Section, from 38 % of women who exhibit a non-reassuring CTG during labour to 25 %, a sample size rounded up to 300 in each group is required (total 600, alpha 0.049 (to allow for one interim analysis), power 90 %). The trial would also have 92 % power to detect a 25 % reduction in combined operative births (caesarean + vacuum + forceps) from 55 % to 41 % [12].

### Compliance

Compliance with the protocol for the trial study groups will be audited by hospital record review of actual attempts to take FBS for lactate estimation in each group. Barriers to compliance will be identified and, where possible, addressed as soon as practical during the trial.

### Feasibility

The Royal Women’s Hospital is the largest tertiary maternity centre in Victoria, Australia, with over 7000 births annually. The investigator team includes the Head of the University Department of Obstetrics and Gynaecology, Neonatologists, maternity team leaders, the Medical and Midwifery Directors of Birth Suite and clinical researchers experienced in the evaluation of techniques for fetal welfare assessment. Several members of the investigator team co-authored the hospital’s Clinical Practice Guidelines for fetal scalp blood sampling in labour.

Two of the investigators (CEE and SPB) have previously conducted an RCT of intrapartum fetal oximetry, which was (i) a novel technology that required considerable clinician expertise; and (ii) was not standard care. These factors were noted as barriers to recruitment, as was the challenge of timing of approach when women were in labour. Despite these barriers, we successfully recruited the full sample of 600 in 3.5 years.

### Analysis

The consultant statistician for the interim analysis will present data to the data monitoring committee in unlabelled study groups. The data management team will remain blinded to group allocation until the final analysis. Analysis will be by intention to treat and all randomised subjects will be included. Demographic information will be summarised for the groups using descriptive statistics, to ensure the groups are comparable. Proportions will be compared by Chi-squared analysis, risk ratios and their 95 % confidence intervals. Continuous variables will be compared by t-tests or their non-parametric equivalent. Logistic regression will compare the between-group difference in caesarean section. Time to event data will be analysed by Kaplan-Meier statistics. Women’s scores from the maternal perceptions questionnaire will be compared for the two groups using Wilcoxon signed rank test.

### Independent data monitoring and safety committee (DSMC)

The DMSC (to be convened) will include as a minimum an experienced researcher, a statistician and other personnel with extensive research experience. The Committee will conduct the interim analysis when 50 % of the sample has been recruited and their outcomes are known. A recommendation to stop or modify the trial will be made to the Steering Committee if: (i) it is beyond reasonable doubt that the probability of finding a difference in caesarean section rates between groups is <0.001; or (ii) serious adverse events are worse in one group than another.

### Personnel

We are an experienced team with a strong track record in fetal welfare assessment, including the only NHMRC-funded Australian multicentre randomised trial of fetal oximetry, with its related Cochrane systematic review [49–52, 59]. Our team includes authors of the Cochrane systematic review of intrapartum lactate, co-authors of the RWH Clinical Practice Guideline for fetal blood sampling, the Medical and Midwifery Directors of Birth Suite, obstetric care team leaders, an Epidemiologist, and Neonatologists with experience in large, international randomised trials.

The Steering Committee will be chaired by CEE and will comprise the experienced team of named investigators, with relevant experience in midwifery, obstetrics, neonatology, and RCTs. The Data Management Team will comprise the Chief Investigators and Associate Investigators and the Clinical Research Midwives. Clinical Research Midwives will conduct the trial on a day-to-day basis, during office hours. A statistician will be contracted to assist in setting up and maintaining the database, providing data to the data monitoring and safety committee and the interim and final analyses. We will contract a Health Economist to conduct the economic analysis of the trial.

## Discussion

This randomised trial will be a world-first. Fetal scalp blood sampling for lactate measurement following identification of a non-reassuring CTG in labour has the potential to reduce caesarean section rates, without compromising fetal or neonatal well-being. Such a result would mean that the addition of FBSLM can substantially reduce the health and financial burden from birth by caesarean section. This outcome has significant worldwide implications for women’s health during and following childbirth. Avoiding a caesarean section in an index pregnancy will influence the health of the mother in future pregnancies and will influence health service provision.

Notwithstanding the cost of this trial, there are substantial economic savings to be realised should the hypothesis of reducing caesarean section rates be supported by the trial result [60]. Australian public maternity hospitals receive about $5,000 from the government for each caesarean section, i.e. twice the amount allocated for a vaginal birth [61]. Even if the results of this trial support the hypothesis and were then translated into practice only partially by Australian maternity centres, the cost of the trial would be covered many times over within a relatively short time period of several years. Worldwide, the cost savings would be of great magnitude.

## Abbreviations

CEA: Cost-effectiveness analysis
CTG: Cardiotocography
FBS: Fetal blood sample
FBSLM: Fetal blood sample for lactate measurement
RCT: Randomised controlled trial
RWH: Royal Women’s Hospital, Melbourne, Victoria, Australia

## References

[1] Lumbiganon P, Laopaiboon M, Gulmezoglu AM, Souza JP, Taneepanichskul S, Ruyan P, et al. Method of delivery and pregnancy outcomes in Asia: the WHO global survey on maternal and perinatal health 2007-08. Lancet. 2010;375(9713):490–9. 10.1016/S0140-6736(09)61870-5 20071021

[2] East CE, Leader LR, Sheehan P, Henshall NE, Colditz PB (2010). Intrapartum fetal scalp lactate sampling for fetal assessment in the presence of a non-reassuring fetal heart rate trace. Cochrane Database Syst Rev..

[3] Althabe F, Belizan JM (2006). Caesarean section: the paradox. Lancet.

[4] Li ZZR, Hilder L, Sullivan EA (2013). Australia's mothers and babies 2011. Perinatal statistics series.

[5] Menacker F, Hamilton BE (2010). Recent trends in cesarean delivery in the United States. NCHS Data Brief.

[6] Macklon NS, Greer IA (1997). The deep venous system in the puerperium: an ultrasound study. Br J Obstet Gynaecol.

[7] Rossen J, Okland I, Nilsen O, EggebÃ¸ T (2010). Is there an increase of postpartum hemorrhage, and is severe hemorrhage associated with more frequent use of obstetric interventions?. Acta Obstet Gynecol Scand.

[8] Jain L, Dudell GG (2006). Respiratory transition in infants delivered by cesarean section. Semin Perinatol.

[9] Al-Zirqi I, Stray-Pedersen B, Forsen L, Vangen S (2010). Uterine rupture after previous caesarean section. BJOG.

[10] Getahun D, Oyelese Y, Salihu HM, Ananth CV (2006). Previous cesarean delivery and risks of placenta previa and placental abruption. Obstet Gynecol.

[11] Smith GC, Pell JP, Dobbie R (2003). Caesarean section and risk of unexplained stillbirth in subsequent pregnancy. Lancet.

[12] East CE. Fetal intrapartum pulse oximetry. St. Lucia, Qld; The University of Queensland (Thesis). 2006.

[13] The American College of Obstetricians and Gynecologists. ACOG Practice Bulletin No. 106: Intrapartum fetal heart rate monitoring: nomenclature, interpretation, and general management principles. Obstet Gynecol. 2009;114(1):192-202. 10.1097/AOG.0b013e3181aef106 19546798

[14] National Institute for Health and Care Excellence (2014). Intrapartum Care: Care of healthy women and their babies during childbirth. National Institute for Health and Care Excellence: Guidance.

[15] Liston R, Sawchuck D, Young D (2007). Fetal health surveillance: antepartum and intrapartum consensus guideline. J Obstet Gynaecol Canada.

[16] Royal Australian and New Zealand College of Obstetricians and Gynaecologists (2014). Intrapartum fetal surveillance : clinical guidelines.

[17] The American College of Obstetricians and Gynecologists. ACOG Committee Opinion. Number 326, December 2005. Inappropriate use of the terms fetal distress and birth asphyxia. Obstet Gynecol. 2005;106(6):1469-1470. 10.1097/00006250-200512000-00056 16319282

[18] Umstad MP (1993). The predictive value of abnormal fetal heart rate patterns in early labour. Aust N Z J Obstet Gynaecol.

[19] Devane D, Lalor J (2005). Midwives' visual interpretation of intrapartum cardiotocographs: intra- and inter-observer agreement. J Adv Nurs.

[20] Palomaki O, Luukkaala T, Luoto R, Tuimala R (2006). Intrapartum cardiotocography -- the dilemma of interpretational variation. J Perinat Med.

[21] Saling E, Schneider D (1967). Biochemical supervision of the foetus during labour. J Obstet Gynaecol Br Commonw.

[22] Jaiyesimi RK, Hickey WP (1990). Fetal haemorrhage after fetal scalp blood sampling. Lancet.

[23] Maiques V, Garcia-Tejedor A, Perales A, Navarro C (1999). Intrapartum fetal invasive procedures and perinatal transmission of HIV. Eur J Obstet Gynecol Reprod Biol.

[24] Pachydakis A, Belgaumkar P, Sharmah A (2006). Persistent scalp bleeding due to fetal coagulopathy following fetal blood sampling. Int J Gynecol Obstet.

[25] Kruger K, Hallberg B, Blennow M, Kublickas M, Westgren M (1999). Predictive value of fetal scalp blood lactate concentration and pH as markers of neurologic disability. Am J Obstet Gynecol.

[26] Westgren M, Kublickas M, Kruger K (1999). Role of lactate measurements during labor. Obstet Gynecol Surv.

[27] Alfirevic Z, Devane D, Gyte GML (2013). Continuous cardiotocography (CTG) as a form of electronic fetal monitoring (EFM) for fetal assessment during labour. Cochrane Database Syst Rev.

[28] Haverkamp AD, Orleans M, Langendoerfer S, McFee J, Murphy J, Thompson HE (1979). A controlled trial of the differential effects of intrapartum fetal monitoring. Am J Obstet Gynecol.

[29] Goodwin TM, Milner-Masterson L, Paul RH (1994). Elimination of fetal scalp blood sampling on a large clinical service. Obstet Gynecol.

[30] Kruger K, Kublickas M, Westgren M (1998). Lactate in scalp and cord blood from fetuses with ominous fetal heart rate patterns. Obstet Gynecol.

[31] Ramanah R, Martin A, Riethmuller D, Maillet R, Schaal JP (2005). Value of fetal scalp lactate sampling during labour: a comparative study with scalp pH. Gynecol Obstet Fertil.

[32] Allen RM, Bowling FG, Oats JJN (2004). Determining the fetal scalp lactate level that indicates the need for intervention in labour. Aust N Z J Obstet Gynaecol.

[33] Engidawork E, Chen Y, Dell'Anna E, Goiny M, Lubec G, Ungerstedt U, et al. Effect of perinatal asphyxia on systemic and intracerebral pH and glycolysis metabolism in the rat. Exp Neurol. 1997;145(2 Pt 1):390–6. 10.1006/exnr.1997.6482 9217075

[34] Myers R, Wagner K, De Courten G (1981). Lactic acid accumulation in tissue as cause of brain injury and death in cardiogenic shock from asphyxia.

[35] Nordström L, Chua S, Roy A, Arulkumaran S (1998). Quality assessment of two lactate test strip methods suitable for obstetric use. J Perinat Med.

[36] Orsonneau J-L, Fraissinet F, Sebille-Rivain V, Dudouet D, Bigot-Corbel E (2013). Suitability of POC lactate methods for fetal and perinatal lactate testing: considerations for accuracy, specificity and decision making criteria. Clin Chem Lab Med.

[37] Reif P, Lakovschek I, Tappauf C, Haas J, Lang U, Schöll W (2014). Validation of a point-of-care (POC) lactate testing device for fetal scalp blood sampling during labor: clinical considerations, practicalities and realities. Clin Chem Lab Med.

[38] Wiberg-Itzel E, Lipponer C, Norman M, Herbst A, Prebensen D, Hansson A, et al. Determination of pH or lactate in fetal scalp blood in management of intrapartum fetal distress: randomised controlled multicentre trial. BMJ (Clinical research ed). 2008;336(7656):1284–7. 10.1136/bmj.39553.406991.25 PMC2413392 18503103

[39] The Royal Women's Hospital. Policy, Guideline and Procedure Manual: Fetal Blood Sampling. *In:* Melbourne, Victoria, Australia; The Royal Women's Hospital. 2014.

[40] Nordstrom L, Ingemarsson I, Kublickas M, Persson B, Shimojo N, Westgren M (1995). Scalp blood lactate: a new test strip method for monitoring fetal wellbeing in labour. Br J Obstet Gynaecol.

[41] Nordstrom L, Ingemarsson I, Persson B, Shimojo N, Westgren M (1994). Lactate in fetal scalp blood and umbilical artery blood measured during normal labor with a test strip method. Acta Obstet Gynecol Scand.

[42] Nordstrom L, Achanna S, Naka K, Arulkumaran S (2001). Fetal and maternal lactate increase during active second stage of labour. BJOG.

[43] Milley JR (1988). Uptake of exogenous substrates during hypoxia in fetal lambs. Am J Physiol.

[44] Westgren M (1998). Lactate compared with pH analysis at fetal scalp blood sampling: a prospective randomised study. Br J Obstet Gynaecol.

[45] Vandenbussche F. Effectiveness of fetal scalp blood sampling for the prevention of cesarean section in case of suspected fetal distress during labor (SCALP-trial): A randomized controlled multicenter study. In: Netherlands trial register. 2013. http://www.trialregister.nl/trialreg/admin/rctview.asp?TC=3837.

[46] Chandraharan E (2014). Fetal scalp blood sampling during labour: is it a useful diagnostic test or a historical test that no longer has a place in modern clinical obstetrics?. BJOG.

[47] Jorgensen JS, Weber T (2014). Fetal scalp blood sampling in labor--a review. Acta Obstet Gynecol Scand.

[48] Schulz KF, Altman DG, Moher D, Group C (2010). CONSORT 2010 statement: updated guidelines for reporting parallel group randomised trials. BMJ (Clinical research ed).

[49] East CE, Brennecke SP, Chan FY, King JF, Beller EM, Colditz PB (2006). Clinicians’ evaluations of fetal oximetry sensor placement in a multicentre randomised trial (the FOREMOST trial). Aust N Z J Obstet Gynaecol.

[50] East CE, Brennecke SP, King JF, Chan FY, Colditz PB (2006). The effect of intrapartum fetal pulse oximetry, in the presence of a nonreassuring fetal heart rate pattern, on operative delivery rates: A multicenter, randomized, controlled trial (the FOREMOST trial). Am J Obstet Gynecol.

[51] East CE, Chan FY, Brennecke SP, King JF, Colditz PB (2006). Women's Evaluations of Their Experience in a Multicenter Randomized Controlled Trial of Intrapartum Fetal Pulse Oximetry (The FOREMOST Trial). Birth.

[52] East CE, Gascoigne MB, Doran CM, Brennecke SP, King JF, Colditz PB (2006). A cost-effectiveness analysis of the intrapartum fetal pulse oximetry multicentre randomised controlled trial (the FOREMOST trial). BJOG.

[53] National Health and Medical Research Council (Australia). Changes to National statement on ethical conduct in human research, 2007. Canberra: NHMRC-publications. 2014.

[54] Drummond MF. Methods for the economic evaluation of health care programmes/Michael F. Drummond … [et al.]. 3rd ed. Oxford: Oxford University Press; 2005.

[55] Macones GA, Goldie SJ, Peipert JF (1999). Cost-Effectiveness Analysis: An Introductory Guide for Clinicians. Obstet Gynecol Surv.

[56] Donoghue D, Cust A, National Perinatal Statistics Unit (Australia) (2000). Australian and New Zealand Neonatal Network, 1998.

[57] Hannah MEMM, Hannah WJMD, Hellmann JMBB, Hewson SBA, Milner RMIS, Willan AP (1992). Induction of Labor as Compared with Serial Antenatal Monitoring in Post-Term Pregnancy: A Randomized Controlled Trial. N Engl J Med.

[58] Sarnat HB, Sarnat MS (1976). Neonatal encephalopathy following fetal distress. A clinical and electroencephalographic study. Arch Neurol.

[59] East CE. Fetal pulse oximetry for fetal assessment in labour. Cochrane Database Syst Rev. 2014;10. 10.1002/14651858.CD004075.pub4 PMC7104297 25287809

[60] Allen VM, O'Connell CM, Farrell SA, Baskett TF (2005). Economic implications of method of delivery. Am J Obstet Gynecol.

[61] Gallego G. New rules for caesarean section. In: Health Policy Monitor. 2008. http://hpm.org/en/Surveys/CHERE_-_Australia/11/New_rules_for_caesarean_section.html.

